# A Highly Sensitive Light-Induced Thermoelastic Spectroscopy Sensor Using a Charge Amplifier to Improve the Signal-to-Noise Ratio

**DOI:** 10.3390/s25030946

**Published:** 2025-02-05

**Authors:** Hanxu Ma, Shunda Qiao, Ying He, Yufei Ma

**Affiliations:** 1 National Key Laboratory of Laser Spatial Information, Harbin Institute of Technology, Harbin 150001, China; mhanxu@126.com (H.M.); shundaqiao@126.com (S.Q.); yinghe@hit.edu.cn (Y.H.); 2 Zhengzhou Research Institute, Harbin Institute of Technology, Zhengzhou 450008, China

**Keywords:** methane (CH_4_) detection, light-induced thermoelastic spectroscopy (LITES), quartz tuning fork (QTF), charge amplifier, minimum detection limit (MDL)

## Abstract

A highly sensitive light-induced thermoelastic spectroscopy (LITES) sensor employing a charge amplifier (CA) is reported for the first time in this invited paper. CA has the merits of high input impedance and strong anti-interference ability. The usually used transimpedance amplifier (TA) and voltage amplifier (VA) were also studied under the same conditions for comparison. A standard commercial quartz tuning fork (QTF) with a resonant frequency of approximately 32.76 kHz was used as the photothermal signal transducer. Methane (CH_4_) was used as the target gas in these sensors for performance verification. Compared to the TA-LITES sensor and VA-LITES sensor, the reported CA-LITES sensor shows improvements of 1.83 times and 5.28 times in the minimum detection limit (MDL), respectively. When compared to the LITES sensor without an amplifier (WA-LITES), the MDL has a 19.96-fold improvement. After further optimizing the gain of the CA, the MDL of the CA-LITES sensor was calculated as 2.42 ppm, which further improved the performance of the MDL by 30.3 times compared to the WA-LITES. Additionally, long-term stability is analyzed using Allan deviation analysis. When the average time of the sensor system is increased to 50 s, the MDL of the CA-LITES sensor system can be improved to 0.58 ppm.

## 1. Introduction

Trace gasses are components of the atmosphere or other media, present in extremely low concentrations. Despite their minimal concentration, these gasses play a pivotal role in specific domains [1,2,3,4,5]. The detection of trace gasses has extensive applications in multiple areas, including climate change research, air quality surveillance, production process regulation, leak identification, and biomedical diagnostics [6,7,8,9,10,11,12,13,14,15]. Consequently, the detection of trace gasses holds tremendous significance [16,17,18,19,20,21,22,23,24,25,26].

**Figure 1:**
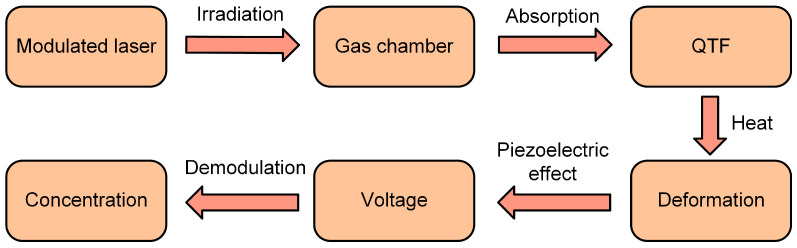
The principle of LITES technique.

Among the various gas detection technologies, spectroscopy-based detection methods are particularly advantageous due to their high sensitivity, strong selectivity, and non-destructive nature [27,28,29,30,31,32,33,34,35,36,37]. In 2002, quartz-enhanced photoacoustic spectroscopy (QEPAS) was first proposed [38]. It employs a quartz tuning fork (QTF) with a narrow response bandwidth and high resonant frequency as the detection components, which can achieve low background noise [39,40,41,42]. This technology has the advantages of low costs, sensitive response, and strong anti-interference ability, and is widely used in gas detection [43,44,45]. However, in QEPAS, the QTF must be in direct contact with the target gas for detection, which limits the use of QEPAS sensors in acidic and corrosive environments. To overcome this limitation, light-induced thermoelastic spectroscopy (LITES) was proposed in 2018 as the second spectroscopic technique based on QTF detectors [46]. The principle of LITES is shown in Figure 1. In LITES, the modulated laser light is absorbed by the target gas in the gas chamber, while the unabsorbed laser light hits the surface of the QTF, causing localized heating and forming an uneven temperature field. The generation of thermal gradient leads to thermal expansion and contraction of the QTF, causing periodic vibration of the QTF and generating piezoelectric signals. By demodulating the electrical signals produced by the piezoelectric effect of the QTF, information about gas concentration can be extracted. The significant advantage of LITES is that it provides a new non-contact measurement technology, which can detect the concentration of the target gas without contact with it. As a result, LITES possesses strong durability and reliability, making it widely used in various gas-sensing applications [47,48,49,50,51,52,53,54,55].

In the LITES sensor system, the electrical signal generated by the piezoelectric effect of the QTF is relatively weak and not easily processed by subsequent signal acquisition devices. Therefore, for the extraction of weak signals, signal amplification is typically achieved through front-end transimpedance amplifiers (TAs) [56,57]. The minimum detection limit (MDL) is closely linked to the signal-to-noise ratio (SNR). Therefore, it is crucial to minimize the noise level of the amplifier as much as possible in order to enhance the sensor’s SNR. To improve the SNR of sensors, some voltage amplifiers (VAs) based on the relatively large voltage constant of the QTF were designed and applied to the field of gas sensing [58,59]. However, the signal source generated by the QTF piezoelectric effect can be equivalent to a high-impedance signal source. When it comes to amplifying high-impedance signal sources, charge amplifiers (CAs) can effectively reduce the noise caused by source impedance and outperform VAs in terms of performance.

This paper reports a highly sensitive, CA-based LITES sensor for the first time. The performance of TA- and VA-based LITES sensors was also studied under the same conditions for comparison. A standard commercial QTF with a resonant frequency of approximately 32.76 kHz was used. By comparing the SNR and MDL of the LITES sensors without an amplifier (WA-LITES) with TA-LITES, VA-LITES, and CA-LITES, the optimal amplifier configuration scheme is selected to further optimize the amplifier gain and evaluate the performance of LITES sensors. Methane (CH_4_) is used as a target gas in these investigations.

## 2. Amplifier Design

**Figure 2:**
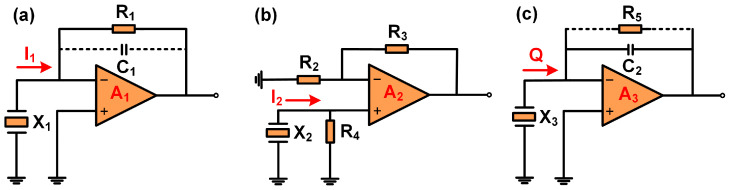
The diagram of three different amplifiers (**a**) TA. (**b**) VA. (**c**) CA.

As a piezoelectric sensing element, the QTF has a relatively high impedance, with typical impedance values ranging from 10 kΩ to 100 kΩ. Consequently, in the design of amplifiers, the QTF is considered a high-impedance signal source. It is conducive to subsequent signal processing that the high-impedance signal source is converted into a low-impedance voltage signal source via a pre-amplifier. In spectroscopic sensors, typically, the amplification of weak signals is accomplished by means of the TA. Given the large voltage constant of the QTF, the VA can also function effectively in this respect. However, when amplifying a high-impedance signal source, the CA can efficiently diminish the noise stemming from the source impedance, appearing to be a preferable option. To select a more optimal amplifier configuration, this paper designs and verifies the performance of LITES sensors, which are, respectively, composed of TAs, VAs, and CAs. The diagrams of three different amplifiers are shown in Figure 2.

Figure 2a illustrates the diagram of the TA. It consists of an operational amplifier (A1), feedback resistor (R1), and QTF (X_1_). Its working principle is as follows: based on Ohm’s law, it converts the input current into an output voltage, and the magnitude of the feedback resistor determines its gain. In LITES sensors, the feedback resistor of the TA is usually set to 10 MΩ to achieve a relatively high gain. However, LITES sensors should fully take into account the parasitic capacitance (C_1_) between the pins of the QTF, which typically ranges from 1 to 2 pF. When the 1 pF parasitic capacitance is connected in parallel with a 10 MΩ feedback resistor, a low-pass filter with a cutoff frequency of approximately 16 kHz will be formed, resulting in significant signal attenuation. In the comparative experiment, R_1_ in the TA was set to 2.7 MΩ.

The VA is composed of an operational amplifier (A_2_), resistor (R_2_), resistor (R_3_), resistor (R_4_), and QTF (X_2_), as shown in Figure 2b. The gain of the VA is related to R_2_, R_3_, and R_4_. Under normal circumstances, the VA can relatively easily obtain a decent gain. In LITES sensors, the QTF sensing element directly amplifies the output piezoelectric signal through the VA, facilitating demodulation by a lock-in amplifier. Compared with the TA, the VA can often achieve more stable and higher gains. However, due to the relatively low input impedance of the voltage amplifier, it is more likely to couple with external noise sources, thus reducing the SNR of VA-LITES sensors. In the comparative experiment, R_2_, R_3_, and R_4_ in the VA were set to 1 kΩ, 10 kΩ, and 24 kΩ, respectively.

The CA utilizes an operational amplifier (A_3_), feedback capacitor (C_2_), and QTF (X_3_) to effectively convert weak charge signals into voltage signals. Its gain is only related to the amount of charge, and the value of the feedback capacitor is independent of frequency. Generally, in order to prevent self-excited oscillation of the CA signal, a resistor (R_5_) is connected in parallel with the C_2_. The diagram of the CA is shown in Figure 2c. In LITES sensors, the signal output by QTF is usually exported via cables, which introduces the influence of additional cable capacitance. However, the output voltage of the CA is only related to the amount of charge and the value of the feedback capacitor and is independent of cable capacitance, which endows the CA with a remarkable ability to suppress irrelevant noise. In addition, the CA also features high input impedance, fast response, and good temperature stability, making it suitable for amplifying the piezoelectric signals generated by the QTF. In the comparative experiment, C_2_ was set to 10 pF in the CA.

**Figure 3:**
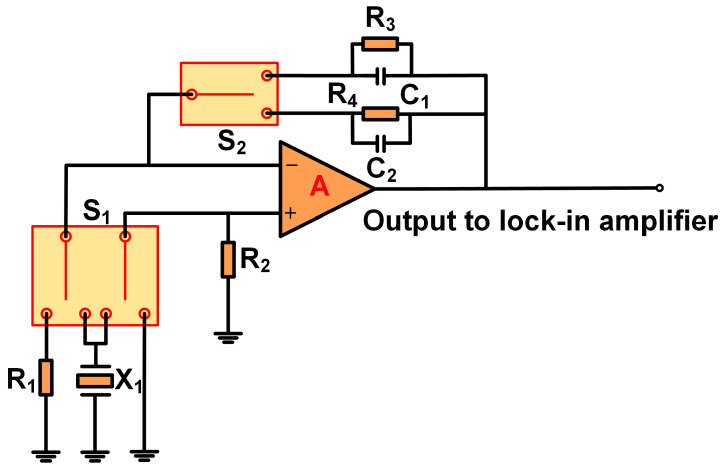
The scheme of composite experimental circuit board for three amplifiers.

The characteristics of the three different amplifiers are listed in Table 1. A composite experimental scheme was designed to verify the performance of the three different amplifiers, as shown in Figure 3. The composite experimental circuit board was fabricated using printed circuit board (PCB) technology. In the designed composite experimental circuit board, different amplifier configurations are achieved by controlling the switches. This design scheme relatively easily realizes switching among the TA, the VA, and the CA, and there is no need to disassemble the amplifier to change the topology, which favorably ensures experiments under the same conditions. In the evaluation of the performance of the three different amplifiers, the AD8034 operational amplifier produced by Analog Devices, Inc. (ADI, Norwood, Massachusetts, USA) was selected.

## 3. Experimental Setup

### 3.1. CH_4_ Absorption Line Selection

**Figure 4:**
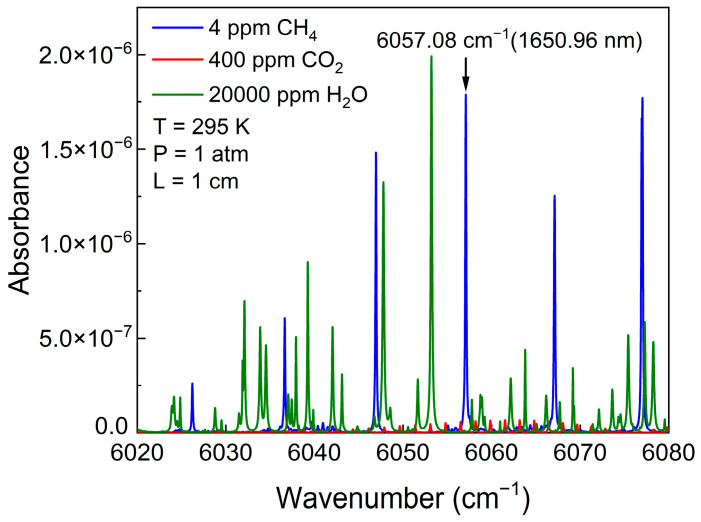
The simulated absorption spectra of different gasses at 6020–6080 cm^−1^.

Gas molecules exhibit unique absorption characteristics at specific frequencies. The selected gas absorption lines should be strong enough and have minimal overlap with those of other gas molecules. Additionally, during the process of choosing gas absorption lines, the experimental cost and feasibility need to be fully considered. Lasers in the near-infrared region are relatively low-cost, and the light sources are stable and reliable. Therefore, we simulated the CH_4_ absorption lines in the range of 6020 cm^−1^ to 6080 cm^−1^. Based on the HITRAN 2020 database, the absorption coefficient of CH_4_ at a temperature of 295 K, a pressure of 1 atmosphere, and an optical path length of 1 cm were simulated, as shown in Figure 4. CH_4_ has several absorption lines, and the strongest one is located at 6057.08 cm^−1^ (1650.96 nm), which is not interfered with by CO_2_ or H_2_O. Thus, this absorption line was selected for the experiment.

### 3.2. The Output Characteristics of DFB Diode Laser

**Figure 5:**
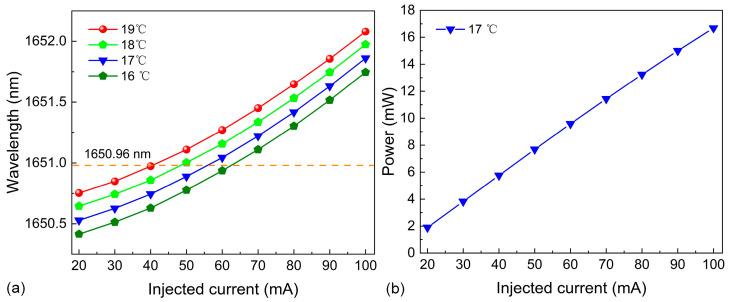
The characteristics of the DFB diode laser. (**a**) Wavelength output characteristics. (**b**) Power output characteristics.

A CW-DFB diode laser with an output wavelength close to 1650 nm was chosen. The wavelength and power of the emitted laser were adjusted by regulating the TEC temperature and injected current, and its performance curves are shown in Figure 5. Figure 5a shows the relationship between the output wavelength of the CW-DFB diode laser and the injected current at temperatures of 16 °C, 17 °C, 18 °C, and 19 °C. When the temperature is set at 17 °C and the injected current reaches 56 mA, the output wavelength of the diode laser is 1650.96 nm, which matches the selected CH_4_ absorption line. The relationship between the output power and the injection current is presented in Figure 5b. At an injected current of 56 mA, the laser output power is close to 8.6 mW.

### 3.3. LITES Sensor Configuration

**Figure 6:**
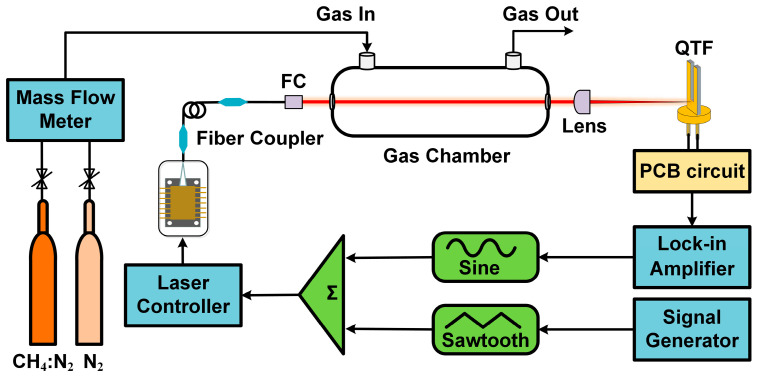
Experimental schematic of the LITES sensor.

Figure 6 depicts the schematic diagram of the LITES sensing system. The laser beam from the pigtail of the diode laser is emitted through a collimator, and then incident into a gas cell with a length of 20 cm to increase the interaction between gas molecules and the modulated laser. After interacting with the gas molecules, the light beam hits the surface of the QTF through a convex lens with a focal length of 50 mm. The surface area of the QTF is rapidly heated, causing the QTF to vibrate. The piezoelectric signal generated by the QTF vibration is amplified through a composite experimental PCB, and then the signal is demodulated using a lock-in amplifier. To suppress background noise, wavelength modulation spectroscopy (WMS) is employed. In wavelength modulation, a high-frequency sine wave signal is superimposed on a low-frequency triangular wave to complete the wavelength modulation of the laser, enabling the laser to tune its wavelength and scan the gas absorption lines. Second-harmonic (2*f*) detection is used, and the 2*f* peak signal is used to infer the gas concentration.

## 4. Results and Discussions

**Figure 7:**
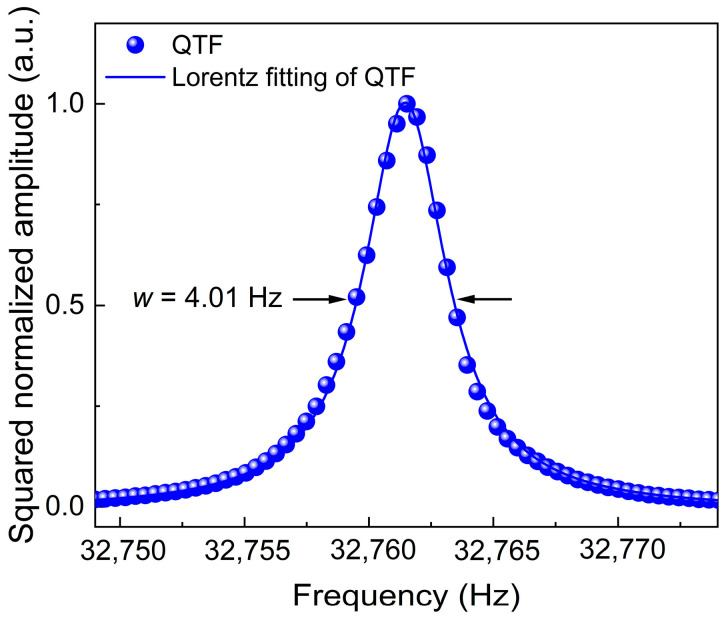
Frequency response characteristics of the used QTF.

The frequency response characteristic of the standard commercial QTF was measured using the optical excitation method. Subsequently, the obtained measurement data were fitted by the Lorentzian function, and the related frequency response curve of the QTF is presented in Figure 7. The resonant frequency of standard commercial QTF was determined to be 32,761.45 Hz, with a response bandwidth (*w*) of 4.01 Hz. The calculated Q value of the QTF is 8169.94.

**Figure 8:**
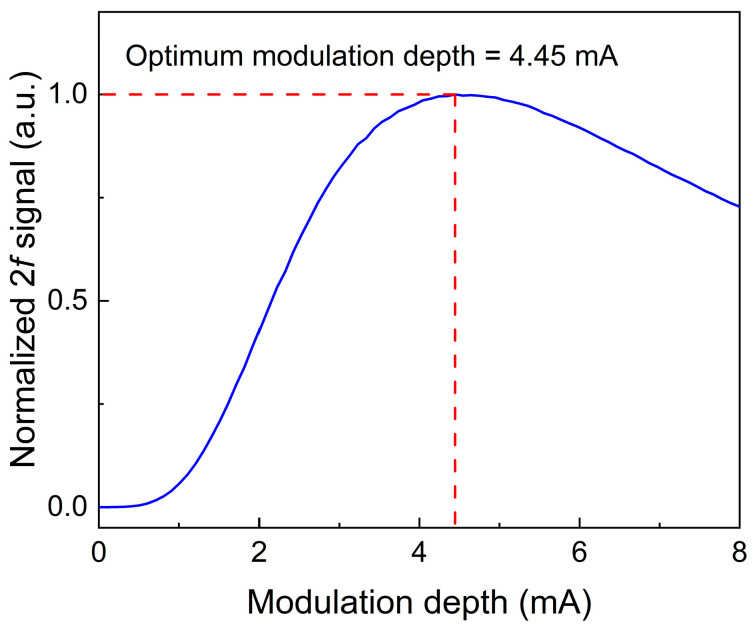
The relationship between the modulation depth and 2*f* signal for the LITES sensor.

The modulation depth of the laser wavelength is of crucial importance in wavelength modulation technology, as it significantly restricts the performance of the LITES sensor system. In order to obtain a stronger thermoelastic signal, the relationship between the modulation depth and the 2*f* signal was studied, and this relationship is shown in Figure 8. As the modulation current increases, the amplitude of the LITES sensor signal first rises and then falls. Therefore, in the subsequent experimental trials, the modulation depth is set to the optimal value of 4.45 mA.

**Figure 9:**
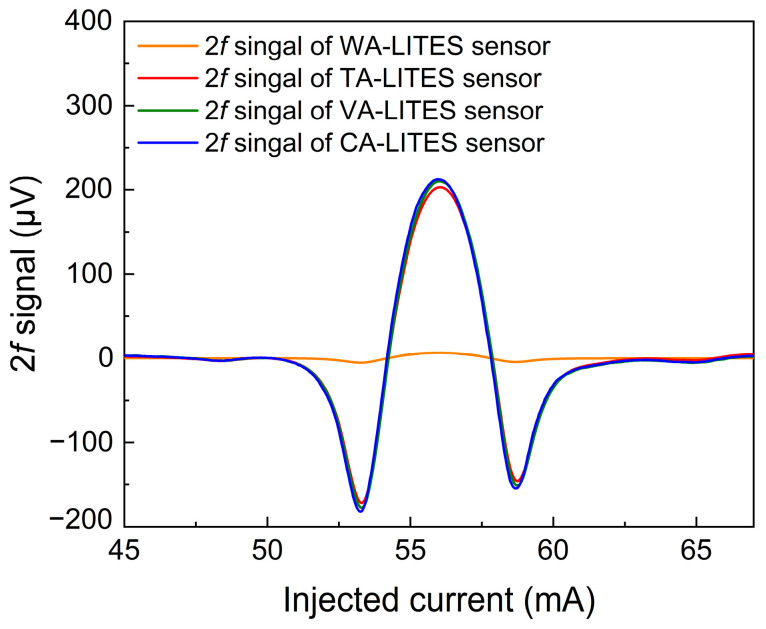
The 2*f* peak signals of WA-LITES, TA-LITES, VA-LITES, and CA-LITES sensors.

After determining the resonant frequency of the QTF and optimizing the modulation current of the laser, the same concentration of methane gas was introduced into the gas chamber, and then the amplification factors of the three amplifiers were adjusted so that they amplified the signals to the same order of magnitude. Then, the 2*f* signals of the WA-LITES sensor, TA-LITES sensor, VA-LITES sensor, and CA-LITES sensor were measured, respectively, as shown in Figure 9. It can be observed that the peak values of the 2*f* signal output of the three amplifiers are all around 210 μV. Such a configuration allows for a more objective performance evaluation of the three amplifiers.

**Figure 10:**
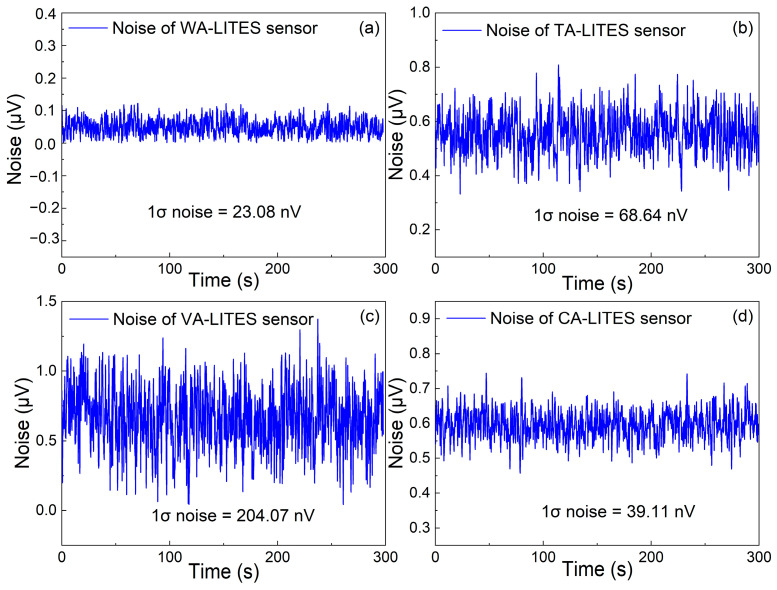
The noise level at pure N_2_ environment. (**a**) WA-LITES sensor. (**b**) TA-LITES sensor. (**c**) VA-LITES sensor. (**d**) CA-LITES sensor.

To evaluate the background noise of the WA-LITES sensor, TA-LITES sensor, VA-LITES sensor, and CA-LITES sensor, respectively, the gas chamber was filled with pure nitrogen (N_2_) at a flow rate of 240 mL/min. The obtained results are shown in Figure 10. The noise standard deviations of the WA-LITES sensor, TA-LITES sensor, VA-LITES sensor, and CA-LITES sensor during the continuous 300-s measurement are 23.08 nV, 68.64 nV, 204.07 nV, and 39.11 nV, respectively, as shown in Figure 10a, Figure 10b, Figure 10c and Figure 10d. 

The peak values of the 2*f* signal, SNR, and MDL of the WA-LITES sensor, TA-LITES sensor, VA-LITES sensor, and CA-LITES sensor are summarized in Table 2. The SNRs for these sensors are 272.33, 2959.04, 1028.35, and 5427.22. The corresponding MDLs are 73.44, 6.75, 19.44, and 3.68. It is evident that, compared to the WA-LITES sensor, TA-LITES sensor, and VA-LITES sensor, the CA-LITES sensor exhibits the best performance, showing a significant advantage in both SNR and MDL. Specifically, the MDL shows improvements of 19.96 times, 1.83 times, and 5.28 times, respectively.

**Figure 11:**
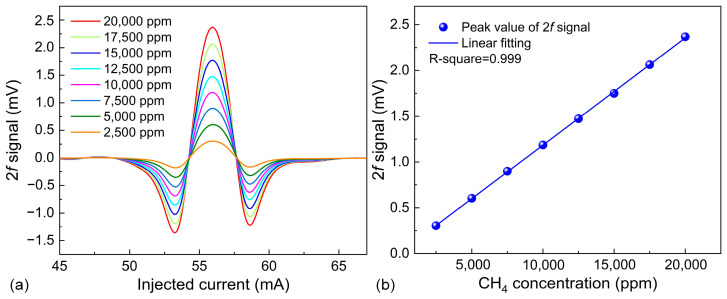
Concentration response for CA-LITES sensor. (**a**) 2*f* signal shapes. (**b**) 2*f* signal peak as a function of CH_4_ concentration.

Based on the experimental comparisons described above, the optimal CA was chosen for subsequent experimentation. To further enhance the performance of the selected CA, the feedback capacitor was configured to 2 pF, and the resistor was configured to 300 kΩ. At this point, the CA gain was increased. Subsequently, the concentration response of the CA-LITES sensor after gain optimization was examined. In this experiment, 2% CH_4_ gas was diluted with pure N_2_ to obtain CH_4_ concentrations ranging from 2500 ppm to 20,000 ppm. The 2*f* signals for different CH_4_ concentrations are shown in Figure 11a. To verify the linear response of the CA-LITES sensor, the peak values of the 2*f* signals at different concentrations were linearly fitted, as shown in Figure 11b. The R-squared value of the fit is 0.999, indicating a strong linear relationship between the concentration and the signal peak in this sensor.

**Figure 12:**
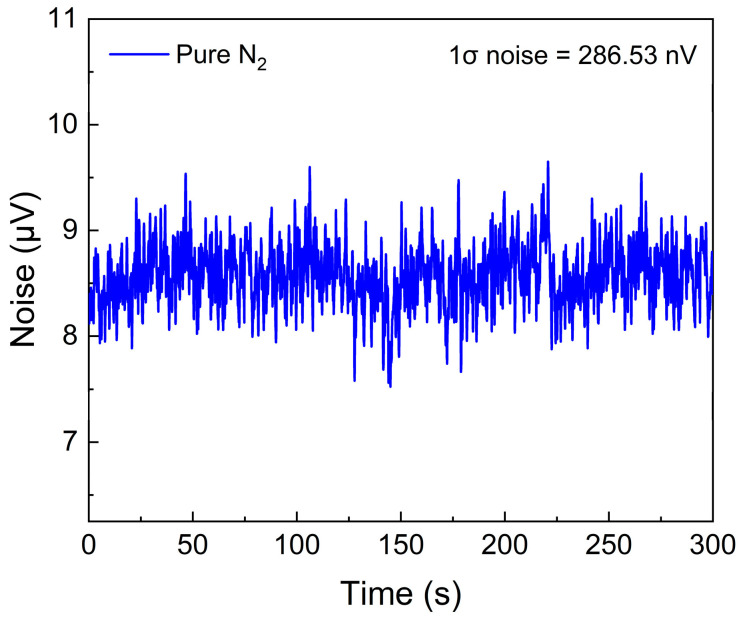
The noise of CA-LITES sensor at pure N_2_ environment after optimizing the gain.

To evaluate the background noise of the CA-LITES sensor after optimizing the gain, pure N_2_ at a flow rate of 240 mL/min was introduced into the gas chamber. As shown in Figure 12, during the continuous 300-s detection, the noise standard deviation of the CA-LITES sensor was 286.53 nV. Therefore, the SNR of the CA-LITES sensor was calculated to be 8263.66. Compared with the WA-LITES sensor, the SNR improved by 30.3 times. The MDL of the CA-LITES sensor was calculated to be 2.42 ppm.

**Figure 13:**
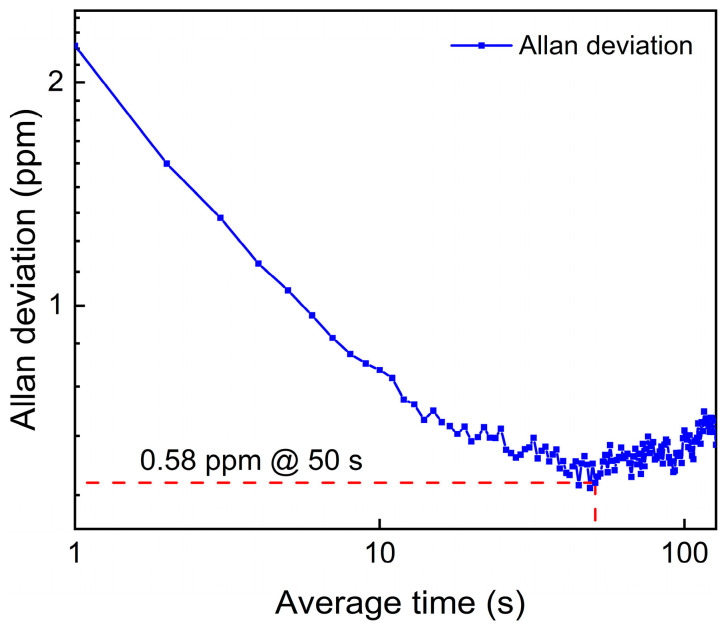
Allan deviation analysis of the CA-LITES sensor.

The long-term stability of the CA-LITES sensor was evaluated through Allan deviation analysis. Continuous measurements were carried out with pure N_2_ for more than three hours. As shown in Figure 13, the Allan deviation initially decreases and then increases as the integration time increases. When the average time of the sensor system increases to 50 s, the MDL of the CA-LITES sensor system can be improved to 0.58 ppm.

## 5. Conclusions

This paper reports a highly sensitive CA-LITES sensor for the first time. The performance of TA- and VA-based LITES sensors was also studied under the same conditions for comparison. A standard commercial QTF with resonant frequency of approximately 32.76 kHz was used. CH_4_ was used as the target gas in these sensors. A CW-DFB diode laser with a center emission wavelength of 1650.96 nm was adopted as the excitation source. Experimental studies have shown that CA has high input impedance and strong anti-interference ability, and its performance is superior to TA and VA. Compared to the WA-LITES sensor, TA-LITES sensor, and VA-LITES sensor, the reported CA-LITES sensor shows improvements of 19.96 times, 1.83 times, and 5.28 times, respectively, in MDL. After further optimizing the gain of the CA, the MDL of the CA-LITES sensor was calculated as 2.42 ppm, which further improved the performance of the MDL by 30.3 times compared to the WA-LITES. In addition, Allan deviation analysis was used to analyze the long-term stability of this CA-LITES sensor. When the average time of the system increases to 50 s, the MDL of the CA-LITES sensor system can be improved to 0.58 ppm.

## Figures and Tables

**Table 1:** Characteristics of three different amplifiers.

Amplifier	Input Signal	Output Signal	Configuration	Output Expression
TA	Current	Voltage	R_1_: 2.7 MΩ	$V_{o}=-I_{1}R_{1}$
VA	Voltage	Voltage	R_2_: 1 kΩ R_3_: 10 kΩ R_4_: 24 kΩ	$V_{o}=-I_{2}R_{4}(1+R_{3}/R_{2})$
CA	Charge	Voltage	C_2_: 20 pF	$V_{o}=-Q/C_{2}$

**Table 2:** The performance comparison of WA-LITES sensor, TA-LITES sensor, VA-LITES sensor, and CA-LITES sensor.

Characteristic	2*f* Peak (μV)	SNR	MDL (ppm)
WA-LITES	6.28	272.33	73.44
TA-LITES	203.12	2959.04	6.75
VA-LITES	209.89	1028.35	19.44
CA-LITES	212.24	5427.22	3.68

## Data Availability

The data presented in this study are available on request from the corresponding author.
